# Upper gastrointestinal endoscopy findings in Mbale Regional Referral Hospital, Eastern Uganda: a 10-year retrospective analysis

**DOI:** 10.4314/ahs.v21i2.54

**Published:** 2021-06

**Authors:** Matthew J Doe, Emmanuel Bua, John SO Obbo, Fred Bisso, Peter Olupot-Olupot

**Affiliations:** 1 Mbale Regional Referral Hospital, P.O. Box 921, Mbale, Uganda; 2 Lira Regional Referral Hospital, Plot 9/19, 21-41 Ngetta Road Police Rd, Lira P.O.BOX 2, Lira, Uganda; 3 Mbale Clinical research Institute, www.mcri.ac.ug, P.O. Box 1966, Mbale Uganda; 4 Busitema University, Faculty of Health Sciences, P.O.Box 1460 Mbale /Mbale Clinical research Institute, P.O. Box 1966, Mbale, Uganda

**Keywords:** Gastrointestinal, OGD, LMIC, oesophageal cancer

## Abstract

**Background:**

Endoscopy is required for formal diagnosis of many upper gastrointestinal (UGI) conditions including oesophageal cancer (OC). There is a paucity of data on endoscopy findings in East Africa as access to testing is challenging for patients. We describe the findings of 10 years of UGI endoscopy in Mbale Regional Referral Hospital (MRRH).

**Method:**

Records of patients that underwent UGI endoscopy in MRRH, November 2009 – March 2019 were retrospectively analysed. Indication, macroscopic findings, histology and patient demographics were retrieved. Sub-group analyses were performed on those with a histological diagnosis of oesophageal cancer.

**Results:**

833 eligible patients received single UGI procedures during the study period. Mean age was 54.8 years, range 16-93 years and 56.9% of patients were male. The main indication was dysphagia (42%) and the most common findings OC (34%) and gastritis (28%). 151 patients had histologically proven OC with a median age of 60 years and a 2:1 male to female ratio. 145/151 (96%) of samples tested revealed squamous cell carcinoma (SCC).

**Conclusion:**

Those undergoing endoscopy in MRRH are most commonly male patients presenting in their 5th decade with dysphagia. There is a high proportion of significant findings including gastritis, peptic ulcer disease, and oesophageal cancer.

## Introduction

Symptoms attributable to the upper gastrointestinal (UGI) tract such as dysphagia, dyspepsia and haematemesis have previously been reported as common in East Africa^1^. The limited investigation capacity and possible lower community awareness may be responsible for lack of data on prevalence, risk factors, early definitive diagnosis, disease progression, and outcomes of UGI pathology in these settings. Persistent symptoms warrant investigation with UGI endoscopy, which is essential for formal diagnosis of important conditions including oesophageal carcinoma (OC) and peptic ulcer disease (PUD).

The World Health Organization indicated that OC is the fifth most common cancer in Uganda^2^. There are areas within East Africa, which border Eastern Uganda, such as Western Kenya^3^ and the Rift Valley^4^ in which OC is considered endemic and may be the most common form of malignancy.

Squamous cell carcinoma (SCC) is the most common histological sub-type in Africa. In contrast, adenocarcinoma remains the most prevalent in some high-income countries within Europe and North America^5^,^6^. In either setting, modifiable risk factors include alcohol intake and smoking^7^,^8^. Previous studies in other parts of Uganda have found a male to female ratio of 2:1^9^. Old age remains a significant risk factor for SCC, but recent data show increasing number of younger people with the condition, suggesting changing epidemiology, especially in endemic areas^10^. Upper GI endoscopy is the only method of obtaining tissue biopsy for formal diagnosis of OC.

Peptic ulcer disease has a high prevalence in East Africa^1^. PUD requires early diagnosis and treatment as complications such as bleeding or perforation can carry significant morbidity and mortality^11^. Risk factors include old age, smoking, use of oral non-steroidal anti-inflammatory medication and Helicobacter pylori (H. pylori) infection^12^,^13^. Prevalence of H.pylori infection in Africa is the highest in the world with up to 70% of people thought to be colonised^14^.

Despite a clear need, endoscopy services in East Africa are limited. In Uganda there is a paucity of data on endoscopy findings^15^. Publications have so far been limited to results from the other three regions of Uganda - Central (Mulago, Kampala)^2^, Western (Mbarara)^16^ and Northern Uganda (Lacor)^17^.

The Eastern region is the second most populous in Uganda with a population of over 9 million. Until now there has been no data published from this region.

This study aims to describe the demographic, presenting symptoms and pathology of patients presenting to Mbale Regional Referral Hospital for UGI endoscopy.

## Methods

### Study Design

A retrospective analysis of all UGI diagnostic endoscopy procedures carried out in Mbale Regional Referral Hospital (Mbale RRH) from 8th November 2009 to 9th March 2019 was performed. This date range was chosen as it captures the full list of procedures from the date the department was opened to present.

### Study Setting

Mbale Regional Referral Hospital (Mbale RRH) is the largest healthcare institution in the Eastern region and houses the region's only endoscopy department. This was established in 2009 with assistance from an international charity (PONT: https://pont-mbale.org.uk). Two local clinicians ran the service until 2018 when they were supported by an international volunteer endoscopist and later a newly trained local surgeon.

### Study Participants

Patients residing in districts served by Mbale RRH who present to primary or secondary care with UGI symptoms are referred to the endoscopy unit by a local clinician. The endoscopy nurse in charge checks their referral for appropriateness, with borderline cases escalated to the endoscopist for a decision. Very occasionally patients procedures are cancelled on the day by the endoscopist if it is felt their symptoms do not warrant UGI endoscopy. A standard fee agreed by hospital management of 75,000 UGX ($20.29 USD) was issued to all patients prior to endoscopy.

### Data Collection

All patients undergoing UGI endoscopy in Mbale RRH are each issued a handwritten procedure report at the time of endoscopy. The department retained copies of all reports during the study period. Where indicated patients underwent tissue biopsy. The samples were fixed in 10% formalin and a histological processing fee of 99,000 UGX ($27.05 USD) was issued. Samples were couriered to Lancet Laboratory in Kampala for histological analysis for those patients able to afford the fee.

### Inclusion/Exclusion Criteria

All records for patients undergoing diagnostic UGI endoscopy during the aforementioned time period were included in the study systematically and sequentially. Procedures were excluded when the handwritten report was illegible, the procedure findings were not specified or when the procedure had to be abandoned without obtaining sufficient views to achieve a diagnosis.

A 7-day endoscopy camp took place in Mbale RRH during January and February 2019. During this 148 diagnostic endoscopy procedures were performed free of charge. Results from this camp have been submitted for publication elsewhere and were excluded from this study.

### Data Entry and Analysis

In March 2019, a research department administrator entered all details from filed reports into a password protected MS-EXCEL database. Information recorded included age, gender, date of procedure, procedure indication (one recorded per patient), procedure diagnosis (up to three recorded per patient), histopathological findings and the name of the endoscopist. The entire database was crosschecked for transcription errors by an endoscopist to ensure quality control.

Statistical analyses were utilised to compare endoscopy findings in male and female patients. The Chi-squared test was used and findings considered significant with p-value <0.05. Oesophageal cancer was considered to be an important diagnosis, made almost exclusively by UGI endoscopy and warranting further evaluation and estimation of prevalence in the Eastern region. Patients diagnosed with OC at the time of endoscopy were thus entered into a more detailed subgroup analysis where histological samples and patient demographic were evaluated.

### Ethical Approval, Consent and Confidentiality

The Mbale Regional Referral Hospital Research & Ethics Committee approved the study. All patients signed a consent form prior to the procedure, the content of which was translated into their own language where possible.

Study data was handled exclusively by endoscopy clinicians and a research administrator from the Mbale Clinical Research Institute. Data was anonymised prior to analysis.

## Results

A total of 862 patients underwent diagnostic UGI endoscopy during the study period at an average of 8 procedures per month. Twenty-nine patient procedures (29/862, 3.4%) were excluded from analysis - nine procedures were incomplete or abandoned and twenty reports were illegible or contained insufficient detail. Single point UGI procedures in 833 patients were included in the study. 474 (56.9%) of included patients were male. The mean age was 54.8 years and range 16 – 93 years. Detailed demographic findings of those presenting for endoscopy are displayed in table 1.

**Table 1:** Demographic of patients presenting for UGI endoscopy

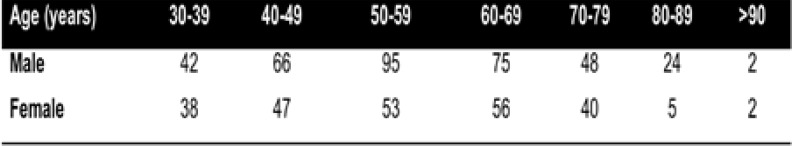

967 findings were recorded for the 833 procedures. We found 120/833 (14.4%) and 14 (1.6%) patients who had two and three diagnoses recorded respectively. Findings for these patients were analysed in the same manner as those with one diagnosis.

**Figure 1:**
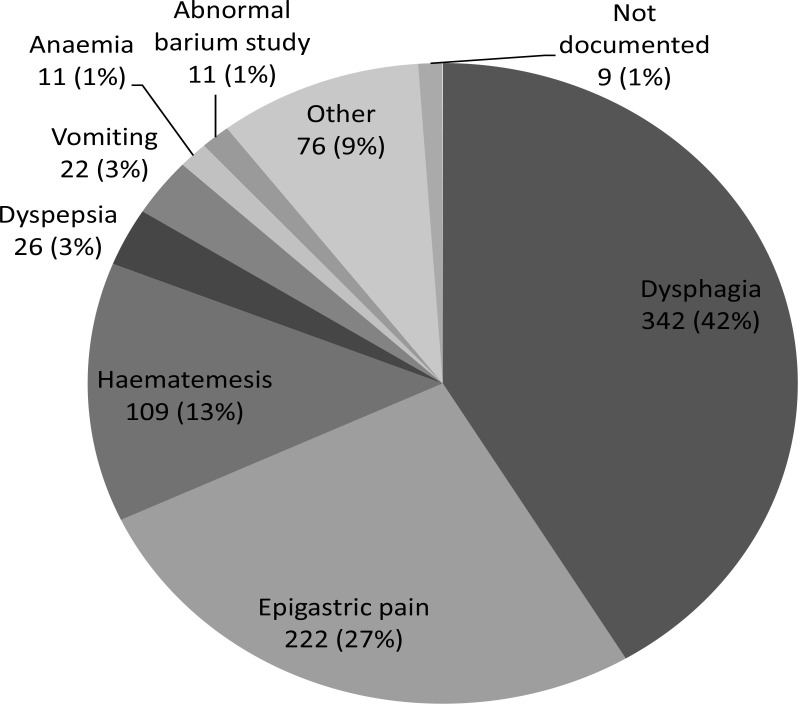
main presenting complaint

Figure 1 illustrates the indications for endoscopy expressed as a percentage and table 2 the indication for endoscopy alongside the most common catagorised findings. Note that one indication and up to three findings were recorded for each patient. Diagnoses made during the study not listed in table 2 are listed in table 3. The most common presenting complaint was dysphagia and the most common diagnosis OC. Of those patients complaining of dysphagia three-quarters (75.7%) were diagnosed with OC at the time of endoscopy. 5.7% of patients had active peptic ulcer disease and 30% had gastritis and/or duodenitis.

**Table 2:** Endoscopy findings correlated to indication (note one indication and up to three findings were recorded for each patient)

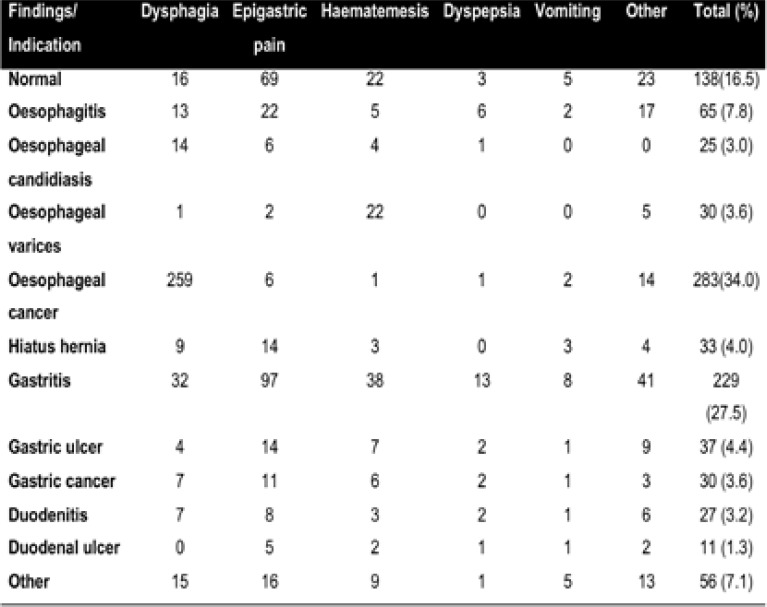

**Table 3:** Other findings (not described in table 1) and their frequency

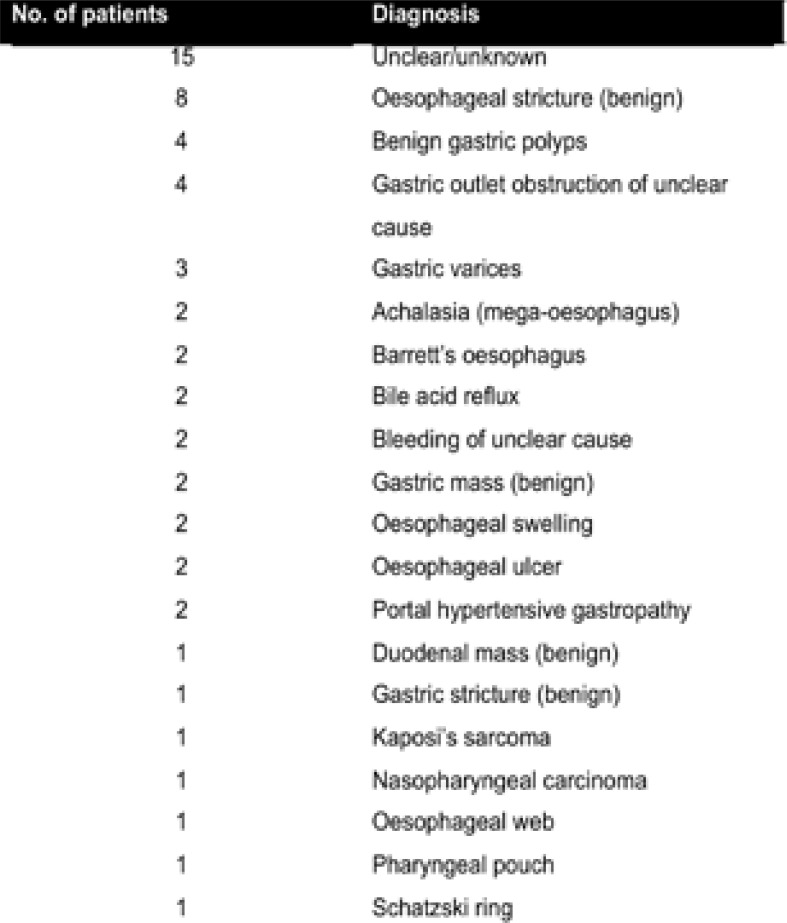

As displayed in table 4, males were significantly more likely to be diagnosed with oesophageal cancer than females. Female patients were considerably more likely to have gastritis or a normal endoscopy than males. No other comparisons reached statistical significance.

**Table 4:** Comparison of endoscopy findings by patient gender

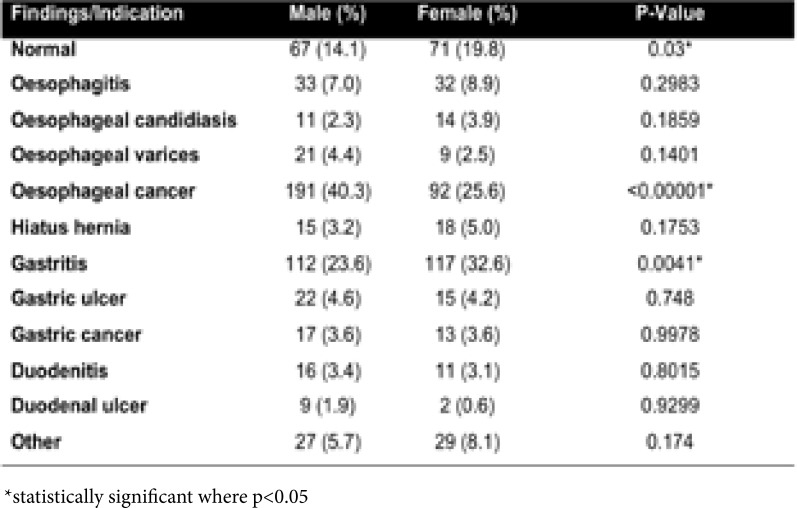

### Oesophageal Cancer

283/833 (34.0%) patients had lesions suspicious of OC at endoscopy, of which a majority 158/283 (56.0%) had biopsy specimens sent for histological analysis. Only 7/158 (4.4%) of these samples did not contain malignant cells on first result.

**Figure 2:**
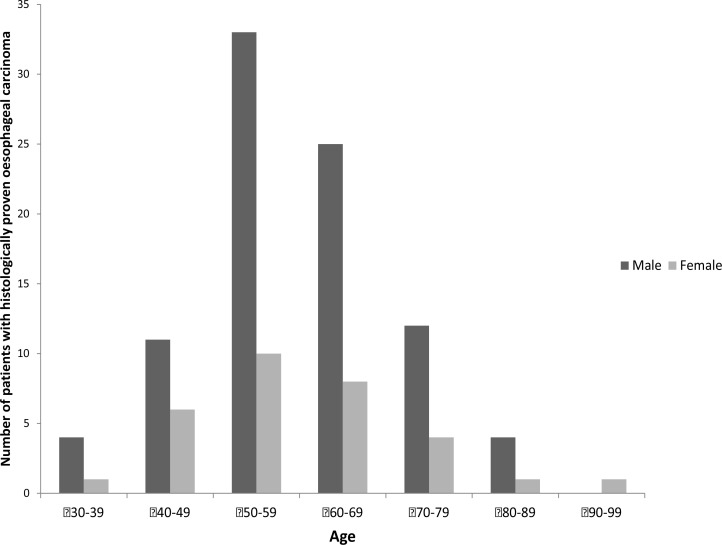
age and gender of those with histologically proven oesophageal carcinoma

Further sub-analysis was performed on the 151 patients with histologically proven OC. The median age was 60 years (standard deviation +/- 11.9 years) and the range 35 – 92 years. 69.5% (105/156) were male. Almost all patients (92%, 139/151) presented with dysphagia, two presented with vomiting and two with epigastric pain. Figure 2 illustrates the number of male and female patients with histologically proven OC in sequential age categories. Of the samples containing malignancy 96% showed squamous cell carcinoma (145/151) and the remaining were adenocarcinoma (6/151).

## Discussion

This study describes the lowest proportion of normal procedures for any study of its kind. A third of endoscopies in our study diagnosed OC – more than double the proportion seen elsewhere^7^,^16^,^17^,^19^. This data represents the highest number of histologically proven OC in any study from Uganda - with 151 cases. Ayuo et al examined 274 cases over a similar time period in Western Kenya, although their study was multi-centre^19^. Nonetheless these results show a comparable male to female ratio to other similar studies performed in East Africa. However, we encountered an older cohort of patients with more significant pathology^7^,^15^,^16^,^17^,^19^. This may be related to delays in accessing services, patient referral systems and population characteristics in this part of Uganda.

Despite catchment areas being comparable, the average number of cases performed per month was fewer in Mbale (8 per month) than other centres in Uganda - such as Mulago (18 per month^7^) and Lacor (86 per month^17^). This suggests that endoscopy is a more limited resource in Eastern Uganda and thus patients are likely to present further into an illness and have a higher likelihood of significant pathology. This may also help to explain why the most common indication for endoscopy was dysphagia, which may be considered a more significant symptom than, for example, dyspepsia. Dysphagia is a more worrying symptom and clinicians are more likely to refer it, while dyspepsia is often treated empirically in the community. Thus they may have been treated with eradication therapy empirically in their communities prior to undergoing endoscopy. Indeed this was the case in a prospective UGI endoscopy study performed in Kampala by Oling et al in 2014^15^. They found that patients had waited more than a year on average for endoscopy since the onset of symptoms, and that 80% had been treated empirically with *H.pylori* eradication therapy prior to endoscopy.

Patients in our study were only accepted for endoscopy by physician referral. The price of an endoscopy procedure was 75,000 Ugandan Shillings and for those undergoing biopsy the histological processing fee was 99,900 Ugandan Shillings. Other study centres have not described their referral system nor the out-of-pocket expense to the patient^4^,^7^,^8^, although Okello et al added a formal outpatient assessment by an endoscopist prior to undergoing a procedure^17^.

Our formal referral system for a comparatively limited service means that potentially only the oldest and sickest patients will be referred for scarce tests by community physicians. Moreover, patients in more affluent study areas such as Kampala may be health conscious and more able to cover the out-of-pocket expenses incurred with UGI endoscopy. They could therefore present with milder symptoms than our population and thus less likely to harbour significant pathology.

Mbale RRH is closer geographically to Eldoret, Kenya than Kampala. One theory regarding OC prevalence is that certain East African tribes may have a genetic predisposition. This would imply that areas closer geographically, regardless of country borders are likely to share demographic data on OC.

While simple demographic data represents a crude method of comparison, our findings do not reflect this theory. Interestingly, the demographic of patients with histologically proven OC was almost identical to that seen in the study performed by Ocama et al in Mulago, Kampala^7^ – both studies showed a median age of 60 years with 69% of cases in males. Elsewhere the median age was anywhere between 55-57 years^3^,^4^,^20^. Unsurprisingly, squamous cell carcinoma was found to be the dominating histological subtype, as found in all studies in Africa^5^.

## Limitations

Where a single endoscopy service and low community awareness for these services exist for a population catchment of many millions, it is difficult to draw reliable conclusions on the prevalence of certain endoscopically diagnosed conditions. This issue is compounded in our study as both the endoscopy procedure and histopathological processing in Mbale RRH incurs a personal financial cost to the patient. Our sample is thus vulnerable to selection bias with those of higher socioeconomic status and/or more significant symptoms undergoing testing and biopsy.

Almost half of our patients with a macroscopically diagnosed malignancy did not have their samples processed by a histopathologist. This could be deemed as an unacceptably high number for a study of this size, however this is reflective of the challenges of a retrospective study in a low-resource setting. Other studies in similar settings have faced the same challenges with cancer specimens.

Limited information was collected on individual patients prospectively, and in some instances the reports were incomplete meaning they had to be excluded. Fortunately the number of reports excluded was low and unlikely to have changed the overall results of the study. Regretfully, we did not collect data on known risk factors for common UGI conditions, such as smoking or alcohol intake, thus were unable to do any in depth analysis of possible aetiology. Only one indication was recorded for each patient, this may have introduced a form of selection bias as patients are likely to report their most significant symptom, or the symptom they believe is most likely to secure them the test. For example, dysphagia was the most common reported indication, however it is likely those with a diagnosis of OC may have suffered significantly with other symptoms such as weight loss and/or odynophagia.

## Conclusion

UGI endoscopy is an essential service even in low-resource settings such as Eastern Uganda. Limited availability of endoscopy means patients are more likely to present or be referred with symptoms of significant morbidity such as dysphagia.

Oesophageal cancer is prevalent in patients presenting for endoscopy to Mbale Regional Referral Hospital, occurs mainly in males and follows a similar demographic pattern to that described in Central Uganda. Squamous cell carcinoma is the most common histological subtype. Where resources are scarce, older patients with dysphagia should be prioritised to receive endoscopy and biopsy.

More studies on endoscopy findings are required in East Africa to further understand the extent of UGI disease. Endoscopy departments throughout Uganda could collaborate to form registries and publish multicentre prospective data in the future. Scale up of endoscopy services is required in the Eastern region to provide greater capacity and encourage more referrals. This will enable earlier diagnosis and treatment of the significant UGI pathology known to be prevalent in the region.

## Declarations

### Availability of data & materials

The datasets used and/or analysed during the current study are available from the corresponding author on reasonable request.
